# NEURAL CORRELATES OF COMPLEX WALKING TASKS IN OLDER ADULTS

**DOI:** 10.1093/geroni/igz038.1760

**Published:** 2019-11-08

**Authors:** Nemin Chen, Caterina Rosano, Helmet Karim, Stephanie Studenski, Howard Aizenstein, Andrea Rosso

**Affiliations:** 1 University of Pittsburgh, Pittsburgh, Pennsylvania, United States; 2 University of Pittsburgh, Department of Epidemiology, Pittsburgh, Pennsylvania, United States

## Abstract

Walking on a narrow path challenges attention and balance control but its neural correlates are unknown. We assessed the association between gray matter microstructural integrity and gait speed along a 6 m long and 20 cm wide path in participants from the Health, Aging, and Body Composition Study (n=155; mean age=83, 53% women, 35% black). Micro-structural integrity was measured by mean diffusivity (MD) in gray matter computed from diffusion weighted imaging (DWI); higher MD indicates lower integrity. We conducted general linear models to assess this association with gray matter microstructural integrity of regions of interest (based on known associations of usual pace gait speed): middle frontal gyrus; caudate; putamen; anterior, middle, and posterior cingulate; hippocampus; precentral gyrus; and supplementary motor area. We adjusted for total brain atrophy, usual pace gait speed, age, sex, race, and education. The average narrow-path gait speed was 0.97 m/s (standard deviation: 0.21). Average usual pace gait speed was 1.1 m/s (standard deviation: 0.21). After adjusting for covariates, we identified significant negative associations between narrow-path gait speed and gray matter MD of left posterior cingulate, left and right hippocampus, and left precentral gyrus (p<0.05). Narrow-path gait speed is associated with lower microstructural integrity in gray matter related to network connectivity (posterior cingulate), spatial cognition (hippocampus), and motor function (precentral gyrus).

